# An attenuated quadruple gene mutant of *Mycobacterium tuberculosis* imparts protection against tuberculosis in guinea pigs

**DOI:** 10.1242/bio.029546

**Published:** 2017-12-14

**Authors:** Ritika Kar Bahal, Shubhita Mathur, Priyanka Chauhan, Anil K. Tyagi

**Affiliations:** 1 Department of Biochemistry, University of Delhi South Campus, Benito Juarez Road, New Delhi 110021, India; 2 Guru Gobind Singh Indraprastha University, Sector 16-C, Dwarka, New Delhi 110078, India

**Keywords:** Multi-gene mutant, BCG, Tuberculosis, Attenuation, Auxotrophic vaccines, Biotin

## Abstract

Previously we had developed a triple gene mutant of *Mycobacterium tuberculosis* (*Mtb*Δ*mms*) harboring disruption in three genes, namely *mptpA*, *mptpB* and *sapM*. Though vaccination with *Mtb*Δ*mms* strain induced protection in the lungs of guinea pigs, the mutant strain failed to control the hematogenous spread of the challenge strain to the spleen. Additionally, inoculation with *Mtb*Δ*mms* resulted in some pathological damage to the spleens in the early phase of infection. In order to generate a strain that overcomes the pathology caused by *MtbΔmms* in spleen of guinea pigs and controls dissemination of the challenge strain, *MtbΔmms* was genetically modified by disrupting *bioA* gene to generate *MtbΔmmsb* strain. Further, *in vivo* attenuation of *MtbΔmmsb* was evaluated and its protective efficacy was assessed against virulent *M. tuberculosis* challenge in guinea pigs. *Mtb*Δ*mmsb* mutant strain was highly attenuated for growth and virulence in guinea pigs. Vaccination with *Mtb*Δ*mmsb* mutant generated significant protection in comparison to sham-immunized animals at 4 and 12 weeks post-infection in lungs and spleen of infected animals. However, the protection imparted by *Mtb*Δ*mmsb* was significantly less in comparison to BCG immunized animals. This study indicates the importance of attenuated multiple gene deletion mutants of *M. tuberculosis* for generating protection against tuberculosis.

## Abstract (precis)

**Summary:** In this study, a mutant of *M. tuberculosis* with the deletion of four important genes has been evaluated in guinea pigs for its attenuation and protective efficacy against tuberculosis.

## INTRODUCTION

*Mycobacterium tuberculosis*, the causative agent of human tuberculosis (TB), continues to be a major cause of mortality. The BCG vaccine provides effective protection against severe forms of TB in children but shows variable efficacy against adult pulmonary tuberculosis (Zodpey and Shrikhande, 2007). The difference in antigenic repertoire of *M. tuberculosis* and BCG leads to the generation of different host immune responses which might be responsible for the limited impact of BCG on control of TB. Live attenuated vaccine strains mimic the natural course of infection and maintain a balance between attenuation and immunogenicity. Several attenuated mutants of *M. tuberculosis* have been tested as TB vaccine strains, only few are able to generate protection equivalent to BCG such as the *panCD*, *cysH*, *proC* and *trpD* mutants of *M. tuberculosis* (Sambandamurthy et al., 2002; Senaratne et al., 2007; Smith et al., 2001). Strains such as Δ*lysA*Δ*panCD* and Δ*leuD*Δ*panCD* demonstrated negligible multiplication in mouse organs, yet generated protection equivalent to BCG (Sambandamurthy et al., 2005; Sampson et al., 2004). On the contrary, their prototype Δ*lysA* and Δ*leuD* strains failed to reduce the bacillary load as much as BCG (Hondalus et al., 2000; Pavelka et al., 2003). Though the attenuated *M. tuberculosis* strains such as MTBVAC (Arbues et al., 2013; Solans et al., 2014; Spertini et al., 2015) and *MtbΔsigH* (Kaushal et al., 2015) have shown promising results, the success rate of TB vaccine in clinical trials is low (Tameris et al., 2013). Thus, novel strains with new combinations of gene deletions need to be evaluated for their potential as vaccine against TB.

We had previously constructed a triple gene mutant of *M. tuberculosis* (*MtbΔmms*), having deletions in genes encoding for phosphatases *mptpA*, *mptpB* and *sapM* that are involved in host-pathogen interaction (Bach et al., 2008; Chauhan et al., 2013; Vergne et al., 2004; Zhou et al., 2010). The mutant *MtbΔmms* demonstrated bacillary growth in the spleens of guinea pigs at 4 weeks post-intradermal administration along with concomitant pathological damage to spleen (Chauhan et al., 2013). Further, animals vaccinated with *MtbΔmms* generated a sustainable and superior protection as compared to BCG in lungs. However, *MtbΔmms* was unable to control hematogenous dissemination of challenge strain to spleen with no significant difference from sham-immunized animals (Chauhan et al., 2013).

In order to overcome the pathology caused by *MtbΔmms* during the early phase of infection, and to generate a strain that controls dissemination of challenge strain, *MtbΔmms* strain was modified to generate an auxotrophic mutant by disrupting *bioA* gene involved in biotin biosynthesis (Attwood and Wallace, 2002; Beckett, 2007; Knowles, 1989; Tang et al., 2014; Mann et al., 2009, 2013). Several studies have demonstrated essentiality of *bioA* for survival of mycobacteria (Keer et al., 2000; Sassetti et al., 2003; Woong Park et al., 2011). We have earlier reported that disruption of *bioA* renders *M. tuberculosis* severely attenuated for growth and virulence in guinea pig along with negligible granulomatous pathology (Kar et al., 2017). Immunization with *MtbΔbioA* imparted significant protection in lungs and spleen when compared to sham-immunized animals demonstrating an efficient control over the dissemination of infecting strain to the spleen (Kar et al., 2017). In this study, we generated a quadruple gene mutant (*MtbΔmmsb*) by disrupting *bioA* gene in *MtbΔmms* strain. Further, we evaluated *in vivo* attenuation and assessed the protective efficacy of *MtbΔmmsb* against virulent *M. tuberculosis* challenge in guinea pigs.

## RESULTS

### Disruption of *bioA* gene in *MtbΔmms*

**Fig. 1.**
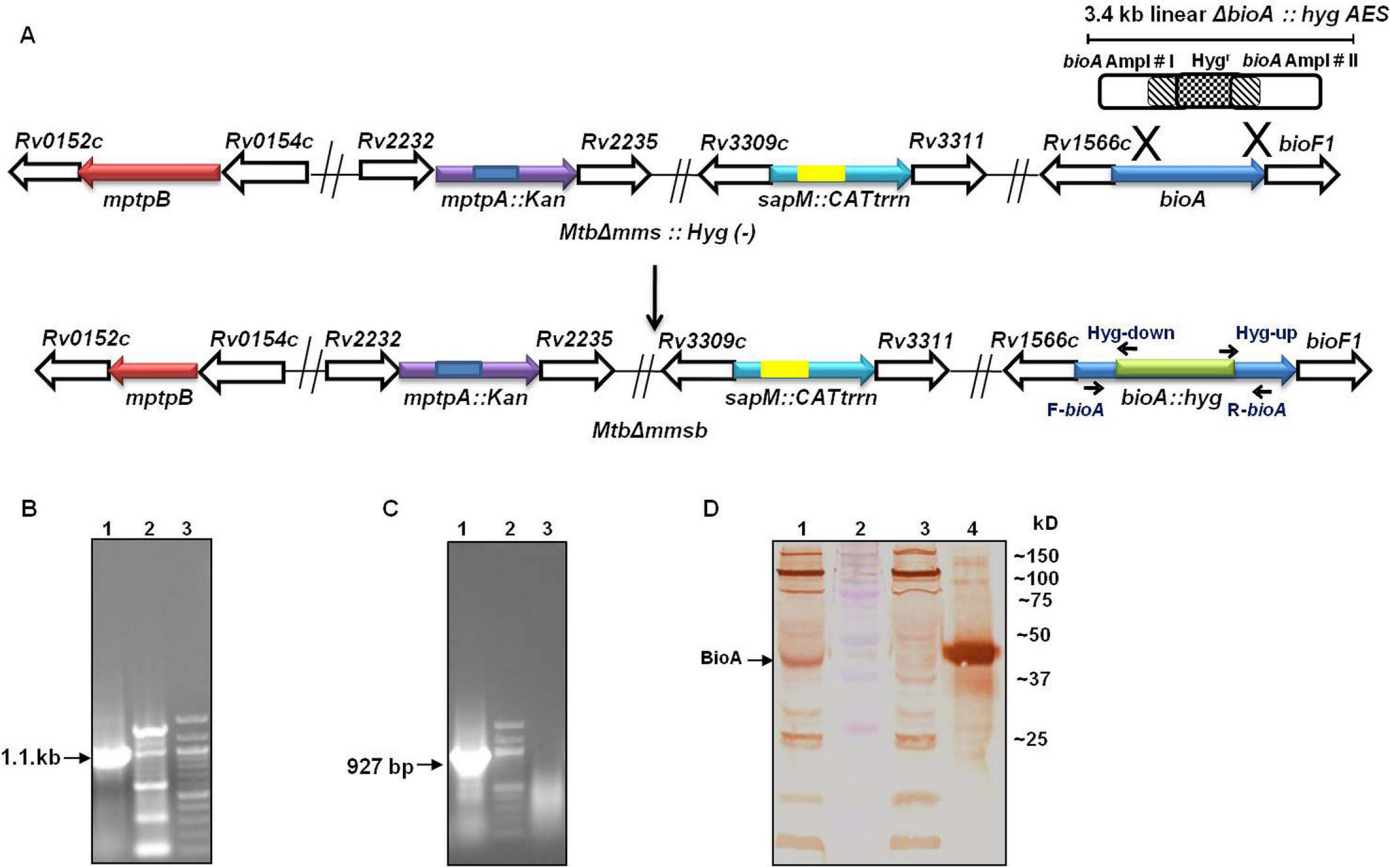
**Disruption of the *bioA* gene in *MtbΔmmsb*.** (A) The figure depicts the disruption of the *bioA* gene in *MtbΔmms* Hyg (−) strain by homologous recombination using *bioA*:hyg AES to generate *MtbΔmmsb*. Arrows show the location of the *bioA* gene-up, *bioA* gene-down, Hyg-up and Hyg-down primers employed for the confirmation of disruption of the *bioA* gene by PCR. (B) PCR-based confirmation of disruption of *bioA* gene in *MtbΔmmsb* by employing *bioA* gene-up and Hyg-down primers. A 1.1 kb PCR amplification product was obtained with the *MtbΔmmsb* genomic DNA as template (lane 1). Lane 2 represents *M. tuberculosis* genomic DNA, and lane 3 represents 100 bp ladder. (C) PCR-based confirmation of disruption of *bioA* gene in *MtbΔmmsb* by employing *bioA* gene-down and Hyg-up primers. A 927 bp PCR amplification product was obtained with the *MtbΔmmsb* genomic DNA as template (lane 1). Lane 2 represents 100 bp ladder and lane 3 represents *M. tuberculosis* genomic DNA. The 1.1 kb and 927 bp PCR products obtained for the *MtbΔmmsb* were confirmed by sequencing. (D) Immunoblot analysis for confirmation of disruption of *bioA* in *MtbΔmmsb*. 10 μg of cell lysate of *M. tuberculosis* (lane 1) and *MtbΔmmsb* (lane 3) were separated on a sodium dodecyl sulfate-polyacrylamide gel (12.5%). Anti-BioA polyclonal antiserum (1:1000 dilution) was used for immunoblot analysis. Expression of *bioA* (∼48 kDa band) was detected in the cell lysate of *M. tuberculosis.* The ∼48 kDa band was absent from the cell lysate of *MtbΔmmsb* confirming the disruption of *bioA* in the mutant. Protein molecular weight marker was loaded in lane 2 and 50 ng of purified *bioA* was loaded in lane 4.

Prior to proceeding with the disruption of the *bioA* gene from *MtbΔmms*, unmarking of hygromycin resistance gene was carried out by employing pYUB870.Gm to obtain *MtbΔmms* (Hyg−) strain (Fig. S1A). Unmarking was confirmed by patching on hyg^−^/hyg+ agar plates (Fig. S1B) and by PCR using *mptpB* internal primers (Fig. S1C). The *M. tuberculosis* strain exhibited an amplification of 391 bp (Fig. S1C, lane 1) while a 2.3 kb PCR product was observed for *Mtb*Δ*mms* (Fig. S1C, lane 3). With unmarked *MtbΔmms* (Hyg−) strain an amplification of 413 bp was observed (Fig. S1C, lane 4, 8). For the construction of *MtbΔmmsb* strain, recombineering method was employed (Fig. 1A). Disruption of *bioA* gene was confirmed by PCR where a *bioA* gene-up and Hyg-down primer pair resulted in an amplification of 1.1 kb in *MtbΔmmsb* (Fig. 1B, lane 1), while an amplification of 927 bp was observed with *bioA* gene-down and Hyg-up primer pair (Fig. 1C, lane 1). The 1.1 kb and 927 bp PCR products obtained for the *MtbΔmmsb* were confirmed by sequencing. Further, immunoblot analysis of *MtbΔmmsb* cell lysate with 1:1000 dilution of polyclonal anti-BioA antiserum (kindly provided by Dr Sabine Ehrt and Dr Dirk Schnappinger, Weill Cornell Medical College, New York) (Woong Park et al., 2011) exhibited absence of ∼48 kDa band, thus confirming the disruption of *bioA* gene from *MtbΔmmsb* (lane 3, Fig. 1D).

### *In vitro* growth kinetics

**Fig. 2.**
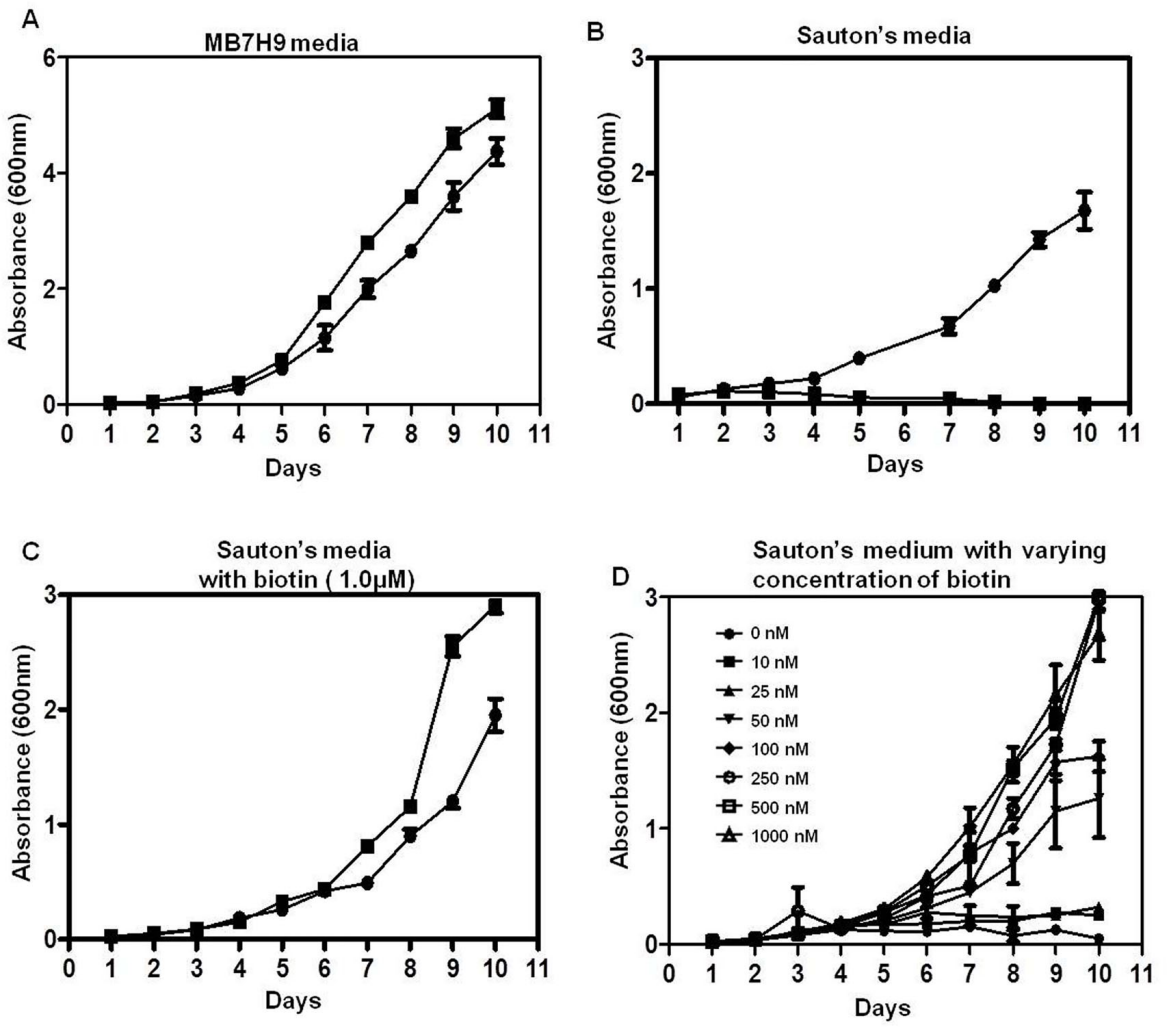
**Characterization of growth of *MtbΔmmsb* in culture media.** Growth of *M. tuberculosis* and *MtbΔmmsb* in (A) MB7H9 medium (biotin rich), (B) Sauton's medium (biotin-deficient medium), (C) Sauton's medium supplemented with 1.0 µM of biotin, and (D) Sauton's medium supplemented with varying concentration of biotin (10 nM-1.0 µM). The growth observed for *MtbΔmmsb* was slightly higher when compared to *M. tuberculosis* after 1 week post-inoculation in media. Growth was monitored for 10 days at 37°C/200 rpm by measuring the absorbance at 600 nm. Closed circle symbols represent data from *M. tuberculosis* strain and closed square symbols represent data from *MtbΔmmsb* strain. Two independent growth analysis experiments were carried out in duplicates and the values of absorbance are represented as the mean (±s.e.m.).

*MtbΔmmsb* strain was able to grow in biotin rich MB7H9 media but it failed to grow in biotin-deficient Sauton's media (Fig. 2A,B). The mutant resumed its growth when biotin was exogenously added in Sauton's media, and growth of *Mtb*Δ*mmsb* was dependent on the amount of biotin added (Fig. 2C,D). Thus, *Mtb*Δ*mmsb* was auxotrophic in nature.

### Attenuation study in guinea pigs

**Fig. 3.**
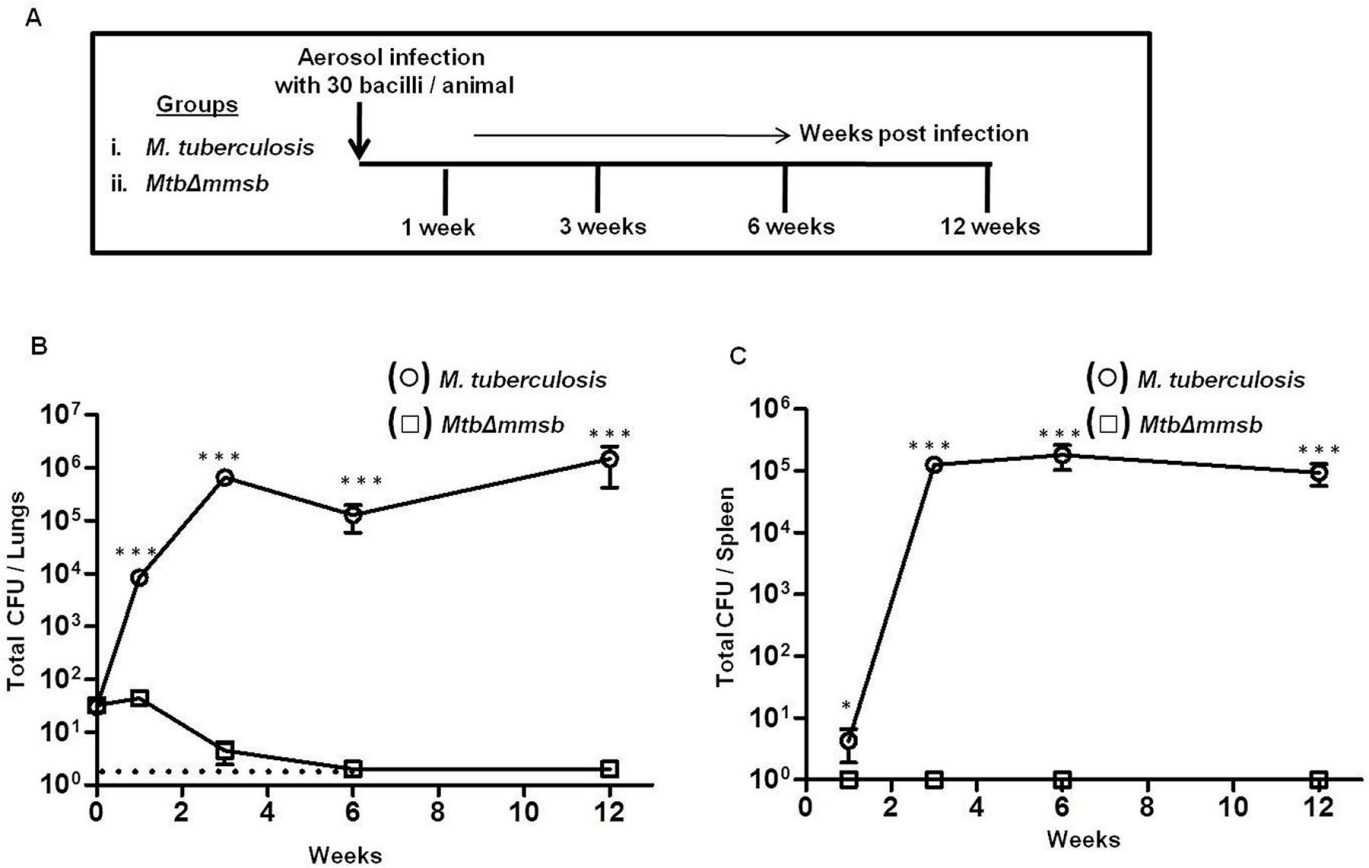
**Attenuation of *MtbΔmmsb* in guinea pigs post aerosol infection.** (A) Experimental protocol for evaluating the attenuation of *MtbΔmmsb*. Guinea pigs were aerosolly infected with ∼30 bacilli of *M. tuberculosis* or *MtbΔmmsb* and euthanized at different timepoints post-infection. (B) Bacillary load in the lungs of guinea pigs (*n*=6) at 1 week, 3 weeks, 6 weeks and 12 weeks post-infection with *M. tuberculosis* (○) or *MtbΔmmsb* strains (□). (C) Bacillary load in the spleens of guinea pigs (*n*=6) at 1 week, 3 weeks, 6 weeks and 12 weeks post-infection with *M. tuberculosis* (○) or *MtbΔmmsb* (□) strains. Each data point represents the total CFU/organ for an individual animal and the bar depicts mean (±s.e.m.) for each group. At 6 weeks and 12 weeks post-infection no bacilli were obtained from the total homogenate of half lung of *MtbΔmmsb*-infected animals. No bacilli were obtained for the complete spleen homogenates of *MtbΔmmsb*-infected animals at any of the time points post-infection. Dotted line represents the limit of detection for bacterial load in lungs. Animals with no bacilli were allotted a total CFU value of 2 in lungs whereas animals with no bacilli in spleen were allotted a total CFU value of 1. *P<0.05, ***P<0.001 (unpaired *t*-test, two-tailed).

Attenuation was evaluated as shown in Fig. 3A. Animals infected with *M. tuberculosis* demonstrated a large increase in the bacillary burden from 1 to 3 weeks post-infection in the lungs and spleen (Fig. 3B and C). By 6 weeks, the bacillary load in *M. tuberculosis* infected animals was stabilized in lungs and spleen and remained high thereafter. As early as 1 week post-infection, *MtbΔmmsb-*infected animals demonstrated significantly lower bacillary load in the lungs when compared to *M. tuberculosis-*infected animals with no detectable bacilli in spleen (Fig. 3B,C). By 3 weeks post-infection the bacillary load further decreased in the lungs of animals infected with *MtbΔmmsb*, and only one animal displayed negligible bacillary load in spleen (Fig. 3B,C). By 6 weeks post-infection no bacilli were detected in the organs of *MtbΔmmsb*-infected animals (Fig. 3B,C).

**Fig. 4.**
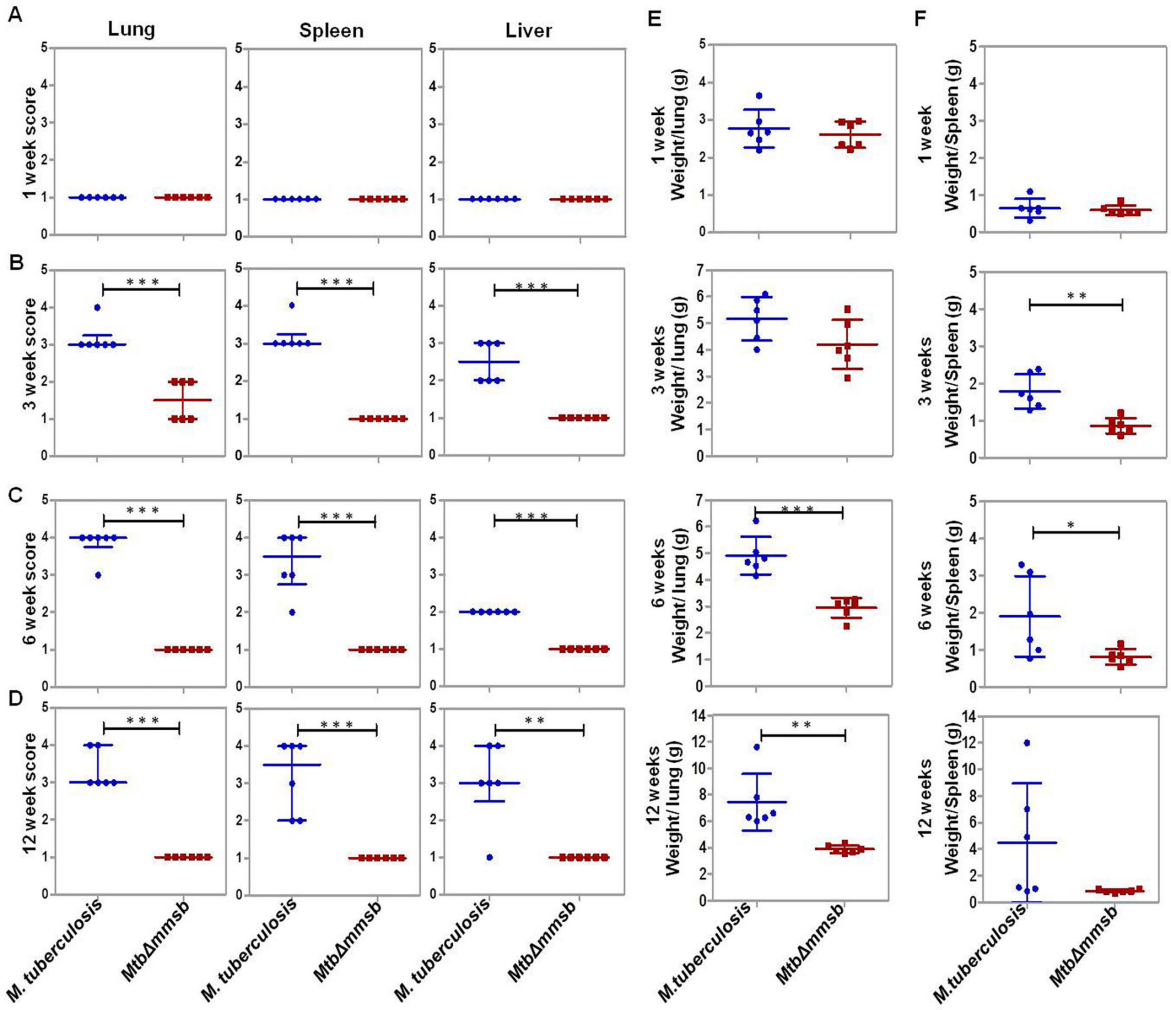
**Gross pathological score and organ weight of animals infected with *M. tuberculosis* or *MtbΔmmsb*.** The figure depicts graphical representation of gross pathological scores assigned at 1 week (A), 3 weeks (B), 6 weeks (C), and 12 weeks (D) to lungs, spleen and liver of guinea pigs following aerosol infection with *M. tuberculosis* or *MtbΔmmsb* strains. Each data point represents the score assigned to an individual animal. The bar depicts median (±interquartile range) for each group. Significant differences were observed for the indicated groups (unpaired *t*-test, two-tailed; **P<0.01 and ***P<0.001). (E,F) The graphical representation of the lungs and spleen weights of guinea pigs at 1, 3, 6 and 12 weeks post aerosol infection. Each data point represents the organ weight of an individual animal and the bar depicts mean (±s.d.) for each group. Significant differences were observed for the indicated groups (unpaired *t*-test, two-tailed; *P<0.05, **P<0.01 and ***P<0.001).

**Fig. 5.**
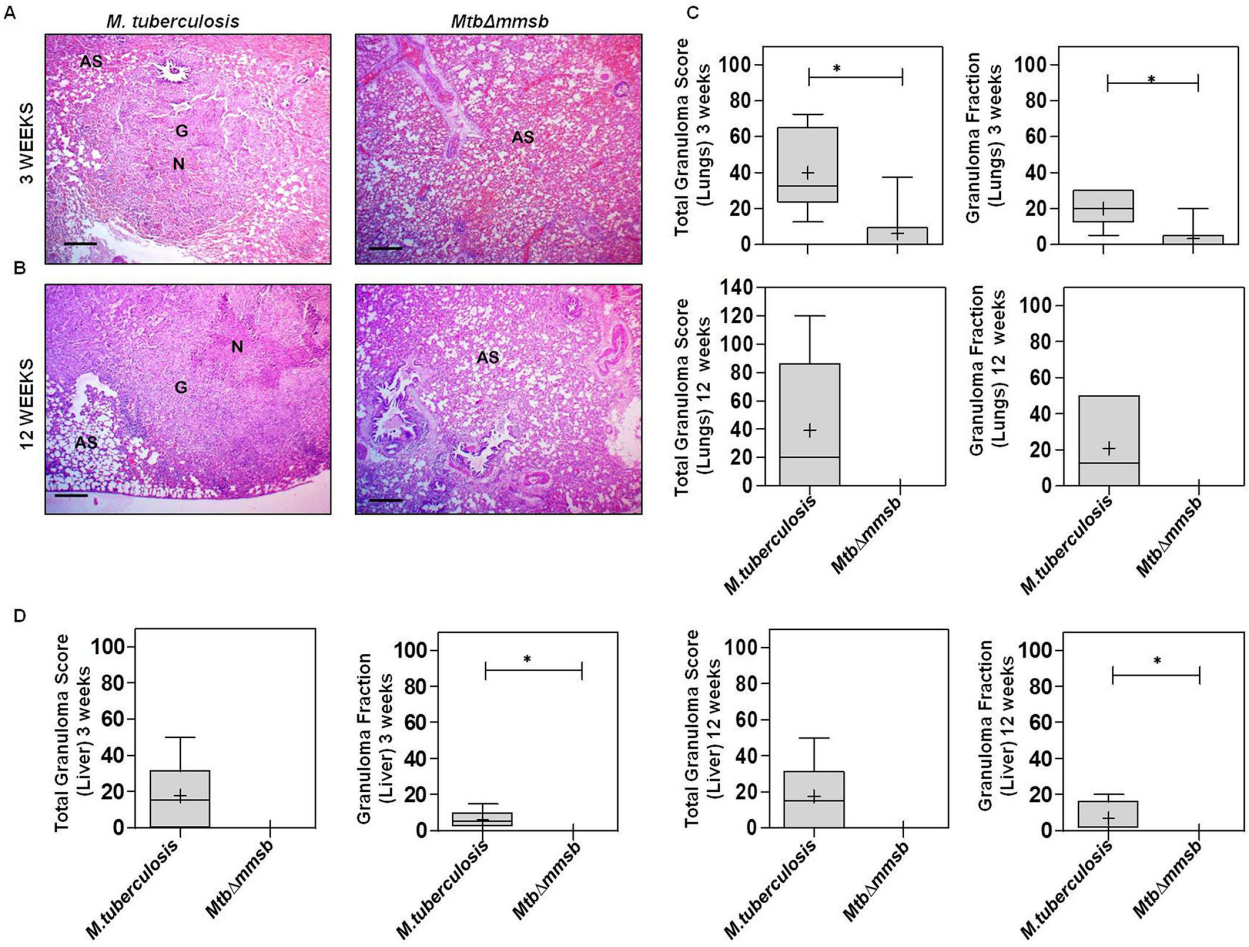
**Histopathological evaluation of organs of guinea pigs infected with *M. tuberculosis or MtbΔmmsb*.** (A) The figure depicts representative 40× magnification photomicrographs of Haematoxylin-Eosin (H&E) stained 5 μm lung sections at (A) 3 weeks and (B) 12 weeks post aerosol infection of guinea pigs infected with *M. tuberculosis* or *MtbΔmmsb.* In this panel N, AS and G denote necrosis, alveolar spaces and granuloma respectively. (C,D) Total granuloma score and granuloma fraction for lungs and liver sections of animals infected with *M. tuberculosis* or *MtbΔmmsb* at 3 and 12 weeks post-infection. Box plots denote the graphical representation of the total granuloma score and granuloma fraction in the lungs and liver sections for each animal per group (box represents the inter quartile range, the minimum and maximum value is denoted by whiskers, median value is denoted by horizontal line and the mean is represented by ‘+’). Scale bar: 300 μm. Significant differences were observed for the indicated groups (Mann–Whitney test, two-tailed; *P<0.05).

*Mtb*Δ*mmsb*-infected animals displayed lesser organ pathology as compared to *M. tuberculosis* infected animals (Fig. S2). At 1 week post-infection both *M. tuberculosis*- and *Mtb*Δ*mmsb*-infected animals exhibited minimal involvement with scanty or no tubercles observed in lungs and spleen (minimum gross pathological score of 1) (Fig. 4A). At 3 weeks post-infection few *Mtb*Δ*mmsb*-infected animals exhibited moderate gross pathological damage of lung with small tubercles occasionally visible, while spleen and liver appeared essentially normal (Fig. 4B). On the other hand, numerous small tubercles were observed in the lungs and liver of animals infected with *M. tuberculosis*, while spleens were moderately enlarged with several small tubercles effacing the entire organ. Importantly, at 6 and 12 weeks post-infection the organs of *Mtb*Δ*mmsb*-infected animals appeared essentially normal with minimal involvement of organs (Fig. 4C,D). On the contrary, *M. tuberculosis*-infected animals exhibited heavy organ involvement with numerous large tubercles, necrotic areas and splenomegaly. Further, *Mtb*Δ*mmsb*-infected animals demonstrated lower mean weight of lung and spleen as compared to the animals infected with *M. tuberculosis* at indicated timepoints, with significant difference observed at 6 and 12 weeks post-infection (Fig. 4E,F). Detailed histopathological analysis was carried out for lung and liver tissues of *Mtb*Δ*mmsb*- and *M. tuberculosis*-infected animals at 3 and 12 weeks post-infection (Fig. 5A). At 3 weeks post-infection significantly less total granuloma fraction was observed for the lungs and liver of *MtbΔmmsb*-infected animals when compared to *M. tuberculosis*-infected animals, which demonstrated numerous necrotic granulomas containing epitheloid cells and lymphocytes (Fig. 5C,D). At 12 weeks post-infection, *M. tuberculosis*-infected animals showed increased granulomatous pathology of lungs and liver while negligible granulomatous pathology was observed for animals infected with *MtbΔmmsb* (Fig. 5C,D). Thus, *MtbΔmmsb* was attenuated for growth and dissemination in the host tissues and could be safely employed as vaccine strain.

### Protective efficacy study in guinea pigs

**Fig. 6.**
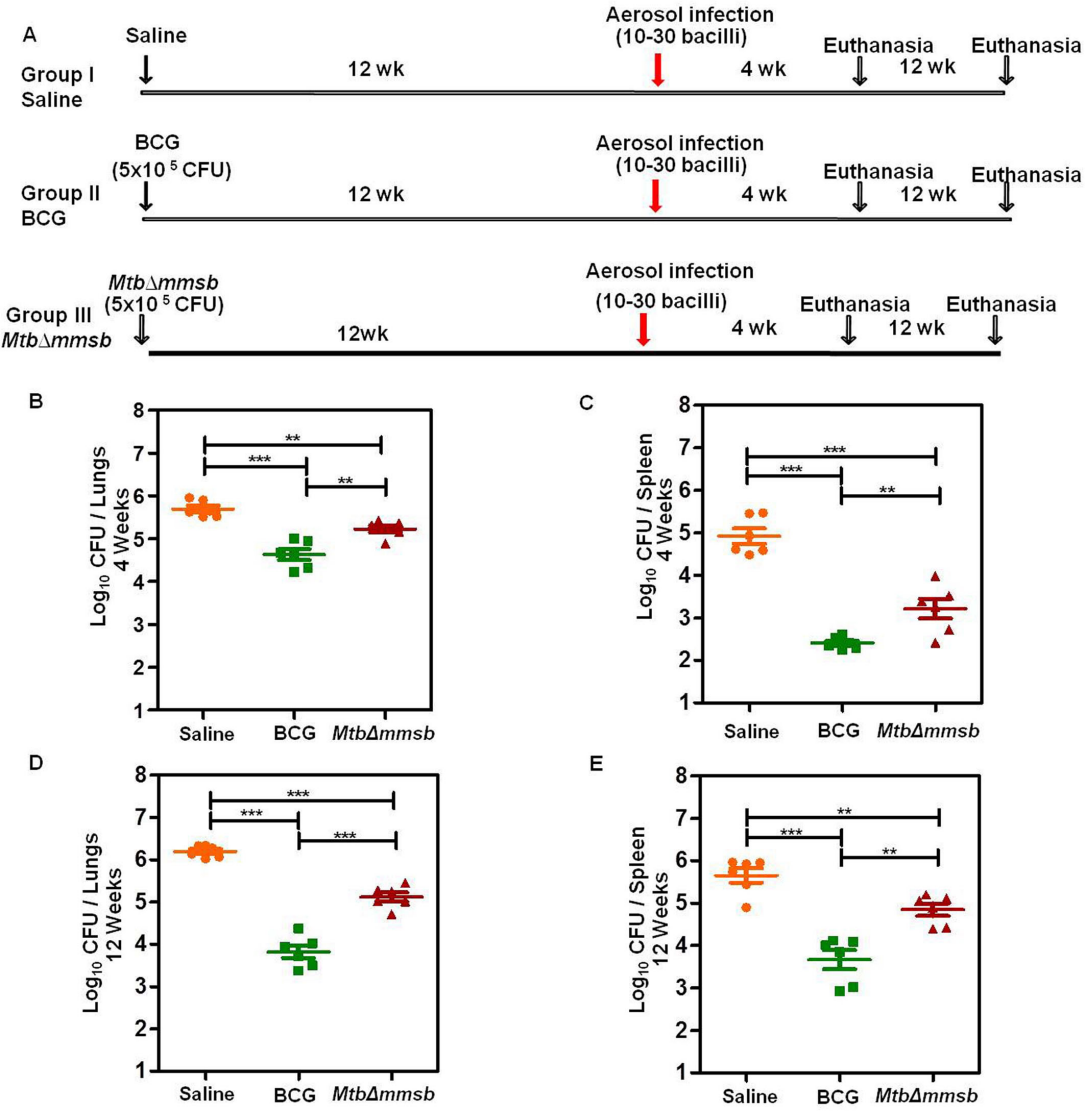
**Assessment of protective efficacy of *MtbΔmmsb* in guinea pig model of experimental tuberculosis.** (A) Experimental protocol for evaluating the protective efficacy of *MtbΔmmsb* against infection with virulent *M. tuberculosis* in guinea pigs. Guinea pigs in groups of 6 were either sham-immunized (group I) or vaccinated with 5×10^5^ CFU of BCG (group II) or with 5×10^5^ CFU of *MtbΔmmsb* (group III). Guinea pigs were challenged with ∼30 bacilli of virulent *M. tuberculosis* via the aerosol route at 12 weeks post primary immunization and euthanized at 4 weeks and 12 weeks post-challenge. (B,C) Bacillary load in the lungs and spleen of vaccinated guinea pigs at 4 weeks post-challenge. Each data point represents the log_10_ CFU/organ for an individual animal and the bar depicts mean (±s.e.m.) for each group. Significant differences were observed for the indicated groups (unpaired *t*-test; two tailed; **P<0.01 and ***P<0.001). (D,E) Bacillary load in the lungs and spleen of vaccinated guinea pigs at 12 weeks post-challenge. Each data point represents the log_10_ CFU/organ for an individual animal and the bar depicts mean (±s.e.m.) for each group. Significant differences were observed for the indicated groups (unpaired *t*-test; two tailed; **P<0.01 and ***P<0.001).

Protective efficacy was evaluated as illustrated in Fig. 6A. At four weeks post-infection, the sham-immunized animals exhibited the highest colony forming units (CFU) in lungs and spleen with bacillary load of 5.69 log_10_ CFU and 4.92 log_10_ CFU, respectively (Fig. 6B,C). In comparison, BCG vaccinated animals exhibited a significantly reduced bacillary load in lungs (by 1.06 log_10_ CFU) and spleen (by 2.51 log_10_ CFU) as compared to the sham-immunized animals. Animals vaccinated with *MtbΔmmsb* also demonstrated ability to control the multiplication and spread of infecting strain with a reduction of bacillary load by 0.46 log_10_ CFU and 1.70 log_10_ CFU in lung and spleen, respectively, when compared to sham-immunized animals (Fig. 6B,C). However, *MtbΔmmsb* failed to provide as much protection as conferred by BCG.

At 12 weeks post-challenge, BCG-vaccinated animals demonstrated significant reduction in bacillary load in lungs by 2.37 log_10_ CFU and in spleen by 1.98 log_10_ CFU when compared to sham-immunized animals (Fig. 6D). Vaccination with *MtbΔmmsb* also imparted significant protection when compared to sham-immunized animals with a reduction of 1.07 log_10_ CFU and 0.80 log_10_ CFU in lungs and spleen, respectively.

Relative to 4 weeks post-challenge, BCG as well as *MtbΔmmsb* showed improved ability to control bacterial multiplication in lungs at 12 weeks post-challenge while both the vaccination strains showed reduced ability to control hematogenous spread to spleen at this timepoint (Fig. 6D,E). However, as seen at 4 weeks, protection generated by *MtbΔmmsb* was significantly less as compared to BCG.

**Fig. 7.**
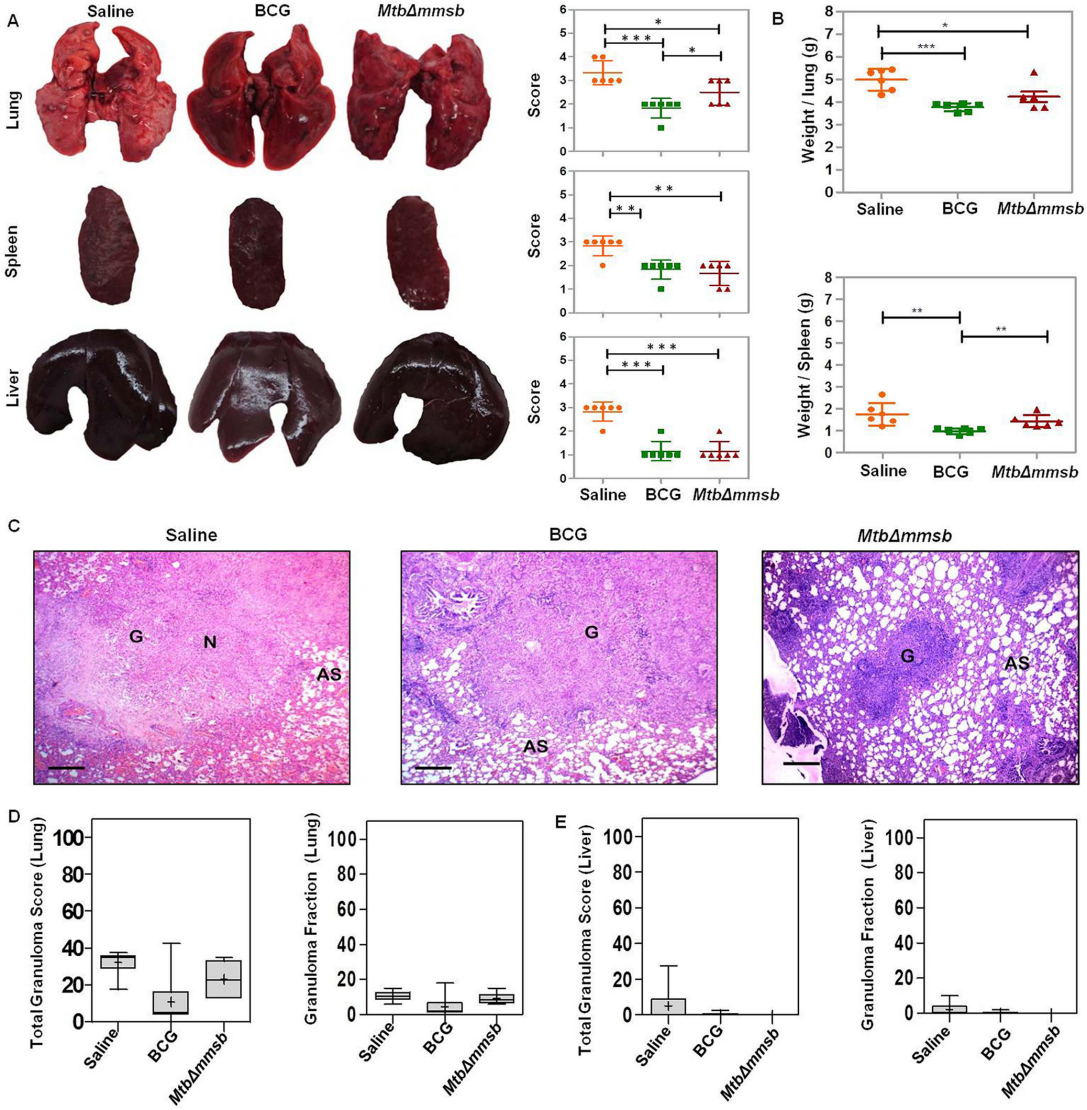
**Histopathological assessment of organs of vaccinated guinea pigs at 4 weeks post-challenge.** (A) Representative photographs of organs (lungs, spleen and liver) of guinea pigs along with the graphical representation of gross pathological scores assigned to the organs of each guinea pig following aerosol infection with *M. tuberculosis* at 4 weeks post-infection. The bar depicts median (±interquartile range) for each group. Each data point represents the score assigned to an individual animal. Significant differences were observed for the indicated groups (unpaired *t*-test, two-tailed; *P<0.05, **P<0.01 and ***P<0.001). (B) The graphical representation of the lung and spleen weights of guinea pigs immunized with saline or BCG or *MtbΔmmsb* at 4 weeks post-challenge. The bar depicts mean (±s.d.) for each group. Each data point represents the organ weight of an individual animal. Significant differences were observed for the indicated groups (unpaired *t*-test, two-tailed; *P<0.05, **P<0.01 and ***P<0.001). (C) Representative 40× magnification photomicrographs of Haematoxylin-Eosin (H&E) stained lung tissue sections of animals immunized with saline or BCG or *MtbΔmmsb* at 4 weeks post-challenge. Scale bar: 300 μm. In this panel N, AS and G denote necrosis, alveolar spaces and granuloma, respectively. (D) Box plots denote the graphical representation of the total granuloma score and granuloma fraction in the lung sections for each animal per group at 4 weeks post-challenge. (E) Total granuloma score and granuloma fraction for liver sections of animals immunized with saline or BCG or *MtbΔmmsb* at 4 weeks post-challenge. Box represents the inter quartile range, the minimum and maximum value is denoted by whiskers, median value is denoted by horizontal line and the mean is represented by ‘+’ (Mann–Whitney test; two-tailed).

**Fig. 8.**
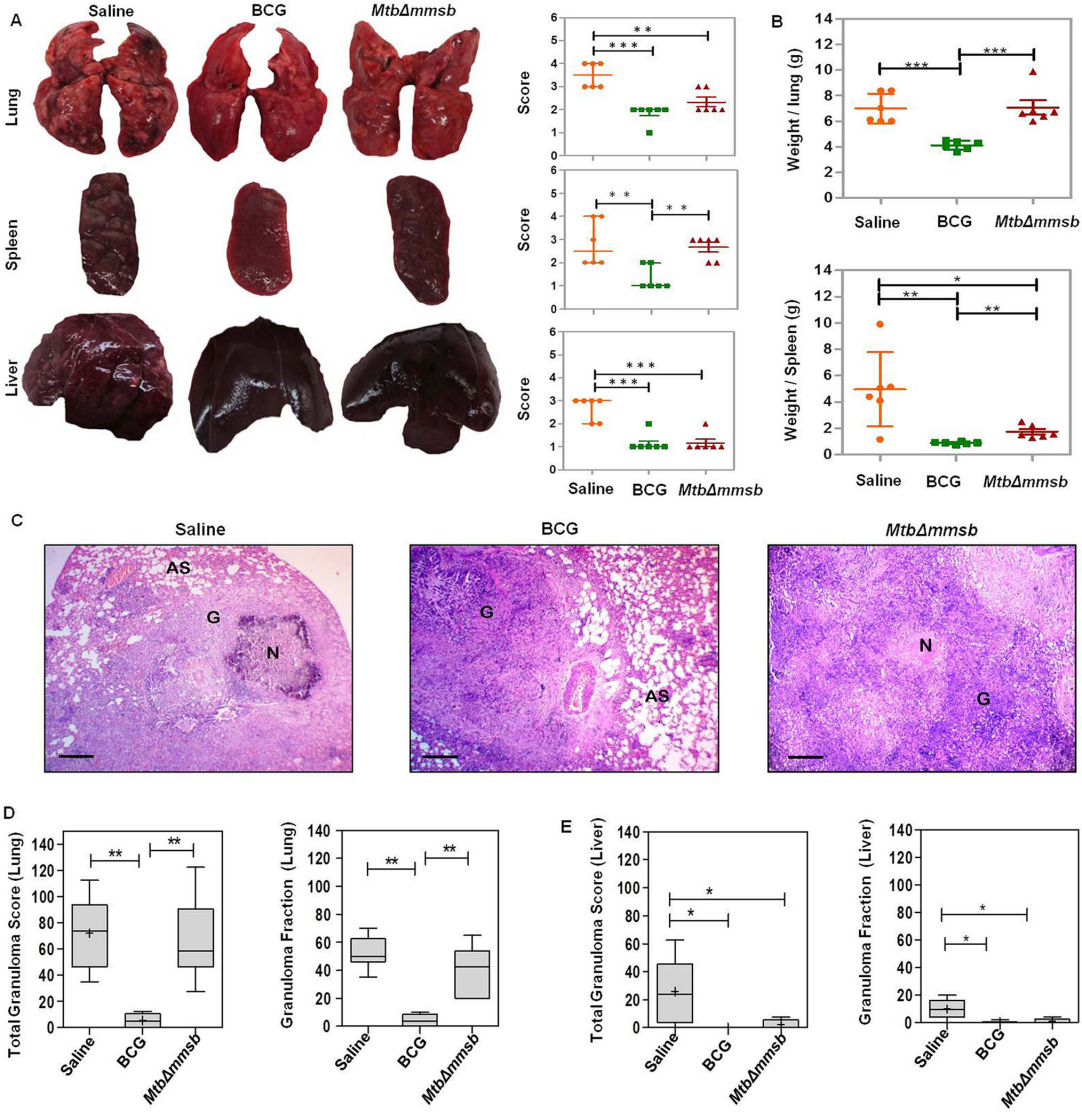
**Histopathological assessment of organs of vaccinated guinea pigs at 12 weeks post-challenge.** (A) Representative photographs of organs (lungs, spleen and liver) of guinea pigs along with the graphical representation of gross pathological scores assigned to the organs of each guinea pig following aerosol infection with *M. tuberculosis* at 12 weeks post-infection. The bar depicts median (±interquartile range) for each group. Each data point represents the score assigned to an individual animal. Significant differences were observed for the indicated groups (unpaired *t*-test, two-tailed; **P<0.01 and ***P<0.001). (B) The graphical representation of the lung and spleen weights of guinea pigs immunized with saline or BCG or *MtbΔmmsb* at 12 weeks post-challenge. The bar depicts mean (±s.d.) for each group. Each data point represents the organ weight of an individual animal. Significant differences were observed for the indicated groups (unpaired *t*-test, two-tailed; *P<0.05, **P<0.01 and ***P<0.001). (C) Representative 40× magnification photomicrographs of Haematoxylin-Eosin (H&E) stained lung tissue sections of animals immunized with saline or BCG or *MtbΔmmsb* at 12 weeks post-challenge. Scale bar: 300 μm. In this panel N, AS and G denote necrosis, alveolar spaces and granuloma respectively. (D) Box plots denote the graphical representation of the total granuloma score and granuloma fraction in the lung sections for each animal per group at 12 weeks post-challenge. (E) Total granuloma score and granuloma fraction for liver sections of animals immunized with saline or BCG or *MtbΔmmsb* at 12 weeks post-challenge. Box represents the inter quartile range, the minimum and maximum value is denoted by whiskers, median value is denoted by horizontal line and the mean is represented by ‘+’. Significant differences were observed for the indicated groups (Mann–Whitney test, two-tailed; *P<0.05 and **P<0.01).

Pathological analysis exhibited that, at 4 weeks post-infection, sham-immunized animals exhibited maximum destruction of lungs, liver and spleen with numerous small or occasional large tubercles spread throughout the organs (Fig. 7A). However, BCG-vaccinated animals displayed significant reduction in gross pathological damage of lungs, spleen and liver with scanty to moderate involvement as compared to sham-immunized animals (Fig. 7A). *Mtb*Δ*mmsb*-vaccinated animals displayed significantly less pathological damage when compared to sham-immunized animals with a moderate number of tubercles effacing the organs. However, lungs of *Mtb*Δ*mmsb*-vaccinated animals demonstrated significantly higher pathological damage as compared to BCG immunized animals (Fig. 7A). Severe pathological damage of organs was observed for sham-immunized animals at 12 weeks post-challenge with lungs and liver exhibiting extensive involvement of tissue with numerous large tubercles, scattered areas of necrosis and occasional splenomegaly (Fig. 8A). BCG-immunized guinea pigs displayed milder pathology in lungs and spleen with smaller granulomas and decreased necrosis while liver exhibited minimal involvement (Fig. 8A). However, at this time point, the animals vaccinated with *MtbΔmmsb* exhibited disorganized lung and spleen phenotype as compared to BCG-vaccinated individuals. The lungs and spleen of animals vaccinated with *MtbΔmmsb* displayed moderate to heavy involvement with numerous small tubercles and necrotic areas while liver tissue appeared similar to BCG immunized animals.

At 4 weeks post-infection, the mean lung weight of *MtbΔmmsb*- and BCG-vaccinated animals was significantly less when compared to sham-immunized animals (Fig. 7B). However, vaccination with *MtbΔmmsb* did not result in any decrease in the weight of spleen from that observed in sham-immunized animals (Fig. 7B). At 12 weeks post-infection the mean spleen weight of *MtbΔmmsb*-immunized animals was significantly less in comparison to sham-immunized animals while there was no difference in mean lung weight (Fig. 8B). BCG-vaccinated animals demonstrated significantly less mean organ weight when compared to sham-immunized as well as *MtbΔmmsb*-vaccinated animals at this timepoint (Fig. 8B).

Histopathologically, at 4 weeks post-challenge, the granuloma score and fraction was highest for lungs and liver of sham-immunized animals which exhibited a large number of necrotic granulomas. Immunization with BCG or *MtbΔmmsb* did not result in any significant difference in the granuloma score or fraction (Fig. 7C-E). At 12 weeks post-infection while sham-immunized animals continued to demonstrate high pathology in the lungs and liver, the lungs of *MtbΔmmsb*-immunized animals also exhibited comparable high granuloma score and fraction (Fig. 8C-E). The lungs of BCG immunized animals exhibited significantly reduced histopathological damage when compared to sham or *MtbΔmmsb* immunized animals (Fig. 8D). However, the liver tissue of BCG- and *MtbΔmmsb*-immunized animals appeared histopathologically comparable and exhibited significantly reduced granuloma score and fraction when compared to sham-immunized animals (Fig. 8E).

## DISCUSSION

Only 5-10% of the individuals exposed to *M. tuberculosis* progress to active disease, indicating that *M. tuberculosis* itself is capable of triggering an effective immune response. Thus, use of live, attenuated *M. tuberculosis* strains as vaccine candidate is a promising strategy for the development of vaccines against TB.

In this study, we have constructed a quadruple gene mutant of *M. tuberculosis* by additional disruption of *bioA* gene in *Mtb*Δ*mms* to generate *Mtb*Δ*mmsb* using recombination through allelic exchange substrate. The disruption of *bioA* gene in the mutant *Mtb*Δ*mmsb* was confirmed by PCR analysis, immunoblot assay and *in vitro* growth characterization. We observed that the growth of the mutant strain was dependent on the concentration of external biotin supplementation which could be rescued with biotin at concentrations as low as 50 nM. The results are in agreement with the previous findings by Woong Park et al. (2011), where little or no growth of *Mtb*Δ*bioA* was observed at concentrations below 25 nM of biotin in media.

Our previous findings have demonstrated that *Mtb*Δ*mms*, the prototype of *Mtb*Δ*mmsb*, showed replication in the spleen during the early phase of infection along with some pathological damage (Chauhan et al., 2013). However, in the present study we found that *Mtb*Δ*mmsb* survived for a very short period and was cleared from the lungs of infected guinea pigs by 6 weeks post-infection with almost no detectable bacilli in spleen. Moreover, while the *M. tuberculosis-*infected animals exhibited severe pathological damage in lung, spleen and liver with disease progression, *Mtb*Δ*mmsb-*infected guinea pigs exhibited negligible organ pathological damage. This implies that disruption of biotin biosynthesis improved the safety profile of multigene mutant. Moreover, ability of some *M. tuberculosis* auxotrophs to provide protection despite high degree of attenuation indicates that even with limited survival in the host, significant immune response can be triggered (Martin et al., 2006; Sakthi et al., 2016; Sambandamurthy et al., 2005; Smith et al., 2001). Nevertheless, further studies are required for evaluating the safety profile of *Mtb*Δ*mmsb* strain in immunocompromised host such as SCID mice.

Upon evaluating the protective efficacy of *Mtb*Δ*mmsb*, we observed that *Mtb*Δ*mmsb* generated significant protection in comparison to sham-immunized animals at 4 and 12 weeks post-infection in lungs and spleen of infected guinea pigs. However when compared to BCG, the protection imparted by *Mtb*Δ*mmsb* was significantly less, which is in contrast to its prototype *Mtb*Δ*mms*, which demonstrated significant protection in lungs. It appears that due to the highly attenuated nature of *Mtb*Δ*mmsb* strain, administration of a single dose may not present certain key antigens to sufficiently trigger a sustained protective response and thus multiple immunizations with *Mtb*Δ*mmsb* may be required. Revaccination with live attenuated vaccines such as BCG is not supported by WHO (Leung et al., 2001). Studies in guinea pigs have also demonstrated that multiple vaccinations with BCG does not improve the efficacy and result in exacerbation of pathology (Basaraba et al., 2006; Moreira et al., 2002). Live attenuated *M. tuberculosis-*based vaccines, when administered in two doses, have demonstrated abrogation of protection generated by a single dose (Kar et al., 2017; Sampson et al., 2004). However, certain live attenuated strains have shown promise in revaccination experiments such as *Mtb*ΔlysA, *Mtb*ΔglnA1 and MTBVAC (Clark et al., 2017; Lee et al., 2006; Pavelka et al., 2003). It will be interesting to test the potential of *Mtb*Δ*mmsb* in a revaccination regimen where *Mtb*Δ*mmsb* is given at a definite time interval with BCG.

The *Mtb*Δ*mmsb* strain needs to be modified for the removal of antibiotic resistance genes employed for its development in accordance with the recommendations on the development of live mycobacterial vaccine candidates in the Geneva consensus (Walker et al., 2010). Additionally, it would be interesting to evaluate the safety profile of the *Mtb*Δ*mmsb* strain when administered via different routes such as intradermally. Also, the efficacy of *Mtb*Δ*mmsb* strain can be evaluated following mucosal administration, as mucosal vaccination is increasingly being recognized as a promising route for immunization against tuberculosis (Kaushal et al., 2015). Further, time to death assay and understanding of immune correlates of protection would emphasize on the future worth of the strain.

### Conclusion

Although preliminary, our findings provide evidence that deletion of genes involved in signal transduction and biotin biosynthesis severely attenuates the pathogen and single immunization with the auxotroph was insufficient for reducing the bacterial burden to levels comparable to BCG. Thus, future studies are focused on testing this multigene mutant as a booster dose in multiple immunization protocols.

## MATERIALS AND METHODS

### Experimental animals

Dunken Hartley guinea pigs (*Cavia porcellus*, female, 250-350 g) were purchased from Disease Free Small Animal House Facility, Lala Lajpat Rai University, India. The animals were housed in individually ventilated cages under standardized conditions in Biosafety level-III facility at University of Delhi South Campus, New Delhi, India and were provided with food and water *ad libitum*. Animals were allowed to acclimatize and were randomized prior to initiation of experiments.

### Ethics statement

Institutional Animal Ethics Committee of University of Delhi South Campus, New Delhi, India, reviewed and approved the guinea pig experiments included in this study (Ref. No. 2/IAEC/AKT/Biochem/UDSC/7.8.2013). All animals were routinely cared for, according to the guidelines of CPCSEA (Committee for the Purpose of Control and Supervision of Experiments on Animals), India, and all efforts were made to ameliorate animal suffering. Animals were intradermally vaccinated by injecting 100 µl of suspension and were euthanized by CO_2_ asphyxiation whenever required during day time in Biosafety level-III facility.

### Bacterial strains and culture conditions

All mycobacterial strains (*M. tuberculosis* Erdman, *M. tuberculosis* H37Rv, *M. bovis* BCG, *Mtb*Δ*mms* and *Mtb*Δ*mmsb)* (Table S1) were grown in Middlebrook (MB) 7H9 broth (BD Difco) supplemented with 1X-ADC (albumin-dextrose–catalase complex, Difco), 0.5% glycerol and 0.05% Tween-80 or on MB7H11 agar (BD Difco) supplemented with 1X-OADC (oleic acid-albumin-dextrose complex, Difco) and 0.5% glycerol. Antibiotics were added at a concentration of 50 µg/ml for hygromycin, 20 µg/ml for gentamicin, 30 µg/ml for chloramphenicol and 25 µg/ml for kanamycin. For vaccination and infection purposes, mycobacterial strains were grown to mid-log phase in supplemented MB7H9 medium. Subsequently, cells were washed with phosphate-buffer saline (PBS), stocks were prepared and stored at −80°C, till further use. The colony-forming unit (CFU) of the PBS stocks was determined by plating appropriate dilutions in duplicates on supplemented MB7H11 agar.

### Construction of *Mtb*Δ*mmsb*

For the generation of quadruple gene mutant, the *bioA* gene was disrupted from the *Mtb*Δ*mms* mutant (Chauhan et al., 2013)*.* The disruptions of *mptpA*, *mptpB* and *sapM* in the *Mtb*Δ*mms* strain were marked by kanamycin, hygromycin and chloramphenicol resistance genes, respectively. Prior to the disruption of *bioA* in *Mtb*Δ*mms*, hygromycin resistance gene was unmarked from the disrupted *mptpB* gene by employing modified helper plasmid pYUB870 (Table S2). The kanamycin resistance gene in pYUB870 was replaced with gentamicin resistance gene to generate pYUB870.Gm which was electroporated into *Mtb*Δ*mms* to generate *Mtb*Δ*mms* (Hyg^−^) strain. Unmarking was confirmed by patching on Hyg^+^/Hyg^−^ agar plates and PCR analysis with *mptpB*-specific primers (Table S2). Next, plasmid pJV53 (Table S2) was employed for the expression of recombineering proteins in *Mtb*Δ*mms* (Hyg^−^) strain to facilitate homologous recombination. The kanamycin resistance gene of vector pJV53 was replaced with gentamicin resistance gene to generate pJV53.Gm which was electroporated into *Mtb*Δ*mms* (Hyg^−^) strain to generate the recombineering strain of *MtbΔmms* expressing gp60/gp61. Subsequently, a 3.4 kb linear Δ*bioA*::*hyg* AES (Kar et al., 2017) was electroporated into *MtbΔmms* (Hyg^−^) and transformants were selected on MB7H11 agar following incubation at 37°C for 3-4 weeks.

### *In vitro* growth analysis

*MtbΔmmsb* and *M. tuberculosis* were grown as described above or in Sauton's media (Himedia) supplemented with 0.5% glycerol and 0.05% Tween-80. Additionally, growth of *MtbΔmmsb* was analysed in Sauton's media supplemented with different concentrations (10 nM to 1000 nM) of biotin (Sigma). The growth kinetics was monitored by measuring A_600nm_ for 10 days.

### *In vivo* attenuation studies

Groups of guinea pigs (*n*=6) were aerogenically infected with either *M. tuberculosis* or *MtbΔmmsb* resulting in an infection dose of ∼30 bacilli in the lungs at day one post-infection and were euthanized at indicated time points post-infection for evaluating bacillary load and pathological damage. Enumeration of bacillary load, gross pathological and histopathological evaluation were carried out as described earlier (Kar et al., 2017).

### Protective efficacy studies

Guinea pigs were intradermally vaccinated with either 100 µl of saline or 5×10^5^ CFU of BCG or 5×10^5^ CFU of *MtbΔmmsb* in 100 µl of saline. Twelve weeks post primary immunization guinea pigs were aerosally challenged with low dose of *M. tuberculosis* H37Rv in an aerosol chamber (A4224 full body Inhalation exposure system, Glas-Col Inc., USA) resulting in 10-30 bacilli in lungs per animal at day one post-challenge. Animals were euthanized at 4 and 12 weeks post-challenge. Enumeration of bacillary load, gross pathological and histopathological evaluation were carried out as described earlier (Kar et al., 2017).

### Statistical analysis

Unpaired *t*-test (two-tailed) was employed for comparison between groups for evaluating bacillary load, organ weight and gross pathological damage of guinea pig organs. Mann–Whitney test (two-tailed) was employed for comparison between groups for analysis of total granuloma score and fraction. Generation of graph and statistical analysis was carried out by employing Prism Software (Graph Pad software Inc., CA). Differences were considered significant when *P*<0.05.

## Supplementary Material

Supplementary information
