# Supplementary material for: An attenuated quadruple gene mutant of Mycobacterium tuberculosis imparts protection against tuberculosis in guinea pigs
Source: Biol Open. 2017 Dec 14;7(1):bio029546. doi: 10.1242/bio.029546 (PMC5829500; doi:10.1242/bio.029546)
Supplement: Supplementary information [file biolopen-7-029546-s1.pdf]

Supplementary Figure. 1

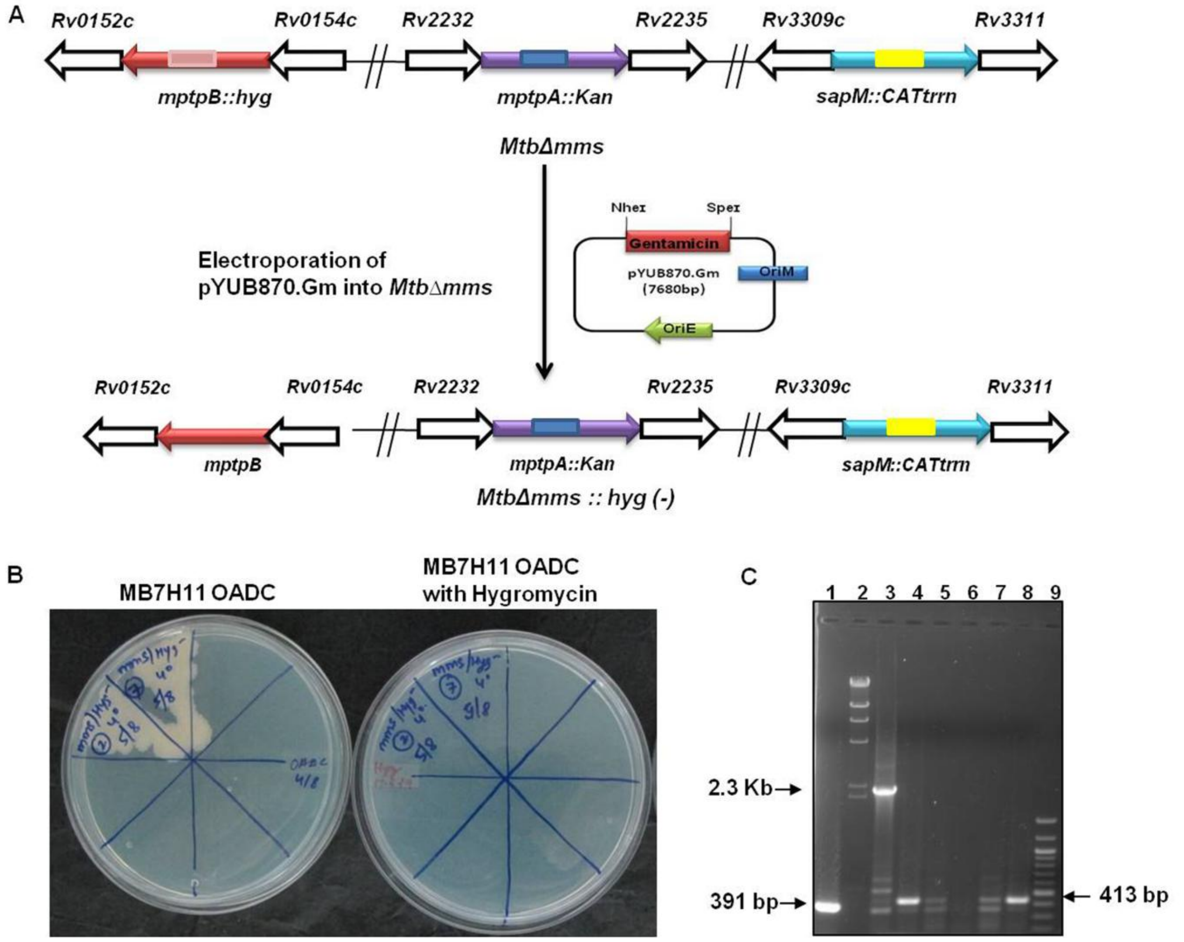

Fig. S1 Unmarking of *MtbΔmms* strain. (a) Diagrammatic representation of the unmarking of hygromycin resistance gene from *MtbΔmms* by employing pYUB870.Gm to generate *MtbΔmms* Hyg (-) strain. The plasmid pYUB870 (8920 bp) was modified by replacing the kanamycin resistance gene with the gentamicin resistance gene in the vector at NheI/SpeI restriction sites to generate pYUB870.Gm (8551 bp). (b) Confirmation of unmarking of hygromycin resistance gene from *MtbΔmms* by patching on agar plates (MB7H11) with or without hygromycin (c) Confirmation of generation of *MtbΔmms* Hyg (-) strain by employing *mptpB* specific primers. Lane 1 represents PCR amplification product of 391 bp obtained with the *M.tuberculosis* genomic DNA as template while lane 3 represents PCR amplification product of 2.3 Kb (104 bp of 5' termini of gene +2 kb hygromycin resistance cassette + 179 bp of 3' termini of gene) obtained with the *MtbΔmms* genomic DNA as template. *MtbΔmms* hyg (-) strain resulted in a 413 bp (104 bp of 5' termini of gene +130 bp res site + 179 bp of 3' termini of gene) PCR amplification product (lane 4

and lane 8). The 413 bp PCR product was sequenced. Lane 2 represents λHindIII molecular marker and lane 9 represents 100 bp ladder.

Supplementary Figure. 2

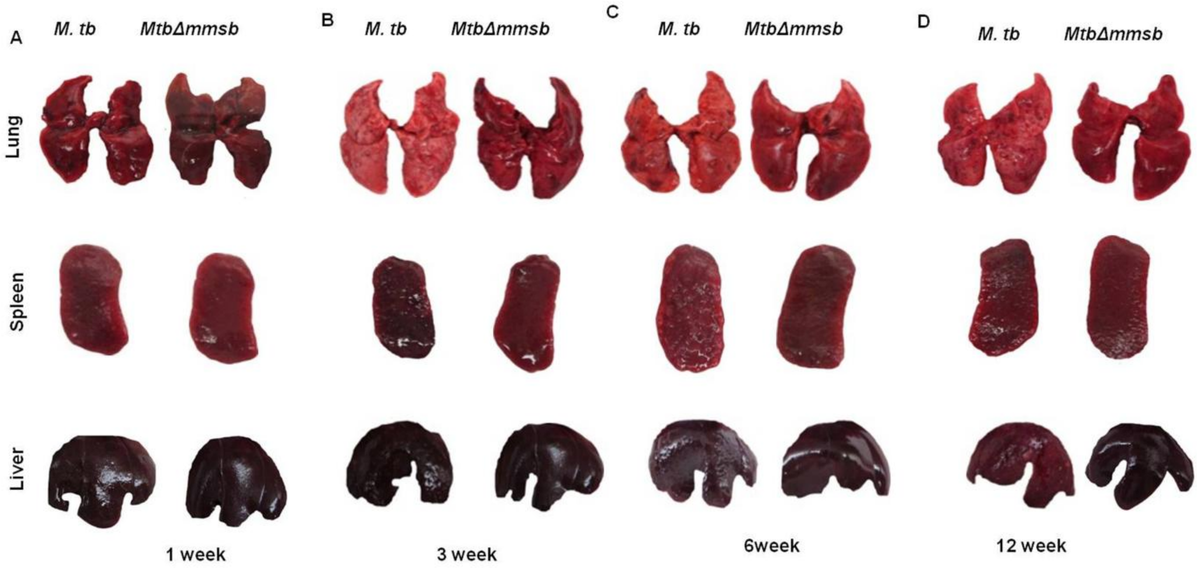

Fig. S2 Gross pathology of organs of guinea pigs post aerosol infection with *M.tuberculosis* or *MtbΔmmsb*. Representative photographs of lungs, spleen and liver of guinea pigs following aerosol infection with *M.tuberculosis (M. tb*) or *MtbΔmmsb* strains at (a) 1 week (b) 3 weeks, (c) 6 weeks and (d) 12 weeks post infection.

Biology Open • Supplementary information

Table S1. Bacterial strains employed in this study

Table S1. Bacterial strains employed in this study		
**Strains**	**Features**	**Reference**
E. coli XL-1 Blue	*endA*1 *gyrA*96 (*nalR*) *thi*-1 *recA*1 *relA*1 *lac glnV*44 F' [::Tn10 *proAB* + *lacIq* Δ (*lacZ*) M15] *hsdR*17 (rK- mK+)	Stratagene, Heidelberg, Germany
E. coli HB101	F-(*gpt-proA*) 62 *leuB*6 *glnV*44 *ara*-14 *galK*2 *lacY*1 (*mcrC-mrr*) *rpsL*20 (Strr) *xyl*-5 *mtl*-1 *recA*13	Life Technologies, CA, USA
M.tuberculosis H37Rv	Virulent strain of *M.tuberculosis*	Dr. J. S. Tyagi, AIIMS, India
M.tuberculosis Erdman	Virulent strain of *M.tuberculosis*	Dr. J. S. Tyagi, AIIMS, India
*Mtb*Δ*mms*	*M.tuberculosis* Erdman mutant harbouring disruption of *mptpA, mptpB* and *sapM* genes	(Chauhan et al., 2013)
*Mtb*Δ*mms* (Hyg-)	*Mtb*Δ*mms* with hygromycin unmarked from its genome	This study
*Mtb*Δ*mmsb*	*M.tuberculosis* Erdman mutant harbouring disruption of *mptpA, mptpB* ,*sapM* and *bioA* genes	This study
M.bovis BCG Danish	Vaccine strain against tuberculosis	BCG laboratories, Chennai, India

Biology Open • Supplementary information

Table S2. Plasmids, primers and antibodies employed in this study

Table S2. Plasmids		
**Plasmids**	**Features**	**Reference**
pYUB870	Helper plasmid carrying tnpR gene employed for the transient expression of Ɣδ- resolvase	(Bardarov et al., 2002)
pYUB870.Gm	Helper plasmid containing gentamicin resistance gene employed for generation of *Mtb∆mms* (Hyg-) strain.	This study
pJV53	Mycobacterial- *E. coli* shuttle vector encoding recombineering proteins gp60 and gp61 from mycobacteriophage Che9c.	(van Kessel and Hatfull, 2007)
pJV53.Gm	Mycobacterial- *E. coli* shuttle vector encoding recombineering proteins gp60 and gp61 with gentamicin resistance marker	This study
pYUB∆*bioA*	pYUB854 with hygromycin resistance cassette flanked with *bioA* amplicons I and II	(Kar et al., 2017)
**Primers**		
F-mptpB	5'gatgatgacgccgacgactcag 3'	This study
R-mptpB	5'gttcggtgtcgaaacgctgctg 3'	This study
BioA Amplicon I up	5′ gatatcactagtatgccaatagcgttcgctga 3′	This study
Hyg down	5'-aaatcagatatccggacaagc-3'	This study
BioA Amplicon I I down	5′ gatatccttaaggcgtcaccacaaccc 3	This study
Hyg Up	5'-cgcacacataaaaacagtgc-3'	This study
**Antibodies**		
Anti-BioA	Polyclonal, Raised in rabbit, specific to BioA	(Woong Park et al., 2011)

Biology Open • Supplementary information
